# Enhancing meningioma tumor classification accuracy through multi-task learning approach and image analysis of MRI images

**DOI:** 10.1371/journal.pone.0327782

**Published:** 2025-08-11

**Authors:** Zahra Mehrpouya, Toktam Khatibi, Abdolazim Sedighipashaki

**Affiliations:** 1 Faculty of Industrial and Systems Engineering, Tarbiat Modares University, Tehran, Iran; 2 Assistant Professor, Department of Radiooncology, School of Medicine Cancer Research Center, Hamedan university of medical sciences, Hamedan, Iran; University of Manitoba, CANADA

## Abstract

**Background:**

Accurate classification of meningioma brain tumors is crucial for determining the appropriate treatment plan and improving patient outcomes. However, this task is challenging due to the slow-growing nature of these tumors and the potential for misdiagnosis. Additionally, deep learning models for tumor classification often require large amounts of labeled data, which can be costly and time-consuming to obtain, especially in the medical domain.

**Objective:**

Our main aim is to enhance Meningioma Tumor Classification Accuracy.

**Method:**

This study proposes a multi-task learning (MTL) approach to enhance the accuracy of meningioma tumor classification while mitigating the need for excessive labeled data. The primary task involves classifying meningioma tumors based on MRI imaging data, while auxiliary tasks leverage patient demographic information, such as age and gender. By incorporating these additional data sources into the learning process, the proposed MTL framework leverages the interdependencies among multiple tasks to improve overall prediction accuracy. The study evaluates the performance of the MTL approach using a dataset of 2218 brain MRI images from 34 patients diagnosed with meningioma, obtained from the Mahdia Imaging Center in Hamadan, Iran.

**Results:**

Results demonstrate that the MTL model significantly outperforms single-task learning baselines, achieving 99.6% ± 0.2 accuracy on the test data in 95% confidence interval.

**Discussion:**

This highlights the efficacy of the proposed approach in enhancing meningioma tumor classification and its potential for aiding clinical decision-making and personalized treatment planning.

**Conclusion:**

Our proposed method can be used in computer-aided diagnosis systems.

## 1. Introduction

A brain tumor is the abnormal and uncontrolled growth of brain cells. The human skull is a rigid and volume-limited structure, which means that any unexpected growth could negatively affect human function, depending on which part of the brain is affected. Additionally, the tumor may spread to other organs and interfere with their function [1]. Around 70% of all brain tumors are considered primary, while the remaining 30% are categorized as secondary. The classification is based on the origin of the tumor. Primary tumors originate in the brain, whereas secondary tumors arise in other parts of the body and subsequently spread to the brain. Typically, secondary tumors are malignant [2].

The most common type of primary tumors occurring in the central nervous system are Meningiomas, accounting for roughly 36% of all cases and 53% of non-malignant tumors. With an incidence rate of 7.86 cases per 100,000 person-years, these tumors are often diagnosed incidentally as they are mostly benign [3]. The World Health Organization classifies meningiomas into three grades, with grade 1 being the most common and benign. However, malignant tumors can arise in 1% to 3% of meningioma cases, resulting in a 5-year survival rate of 32% to 64% [4].

Meningioma is a type of tumor that can be challenging to diagnose as it tends to grow slowly, and its symptoms can be subtle or mistaken for other health conditions [5]. Often, these symptoms are ignored as they can be perceived as normal signs of aging. To diagnose meningioma, a neurologist must conduct a complete neurological examination followed by an imaging test. The recommended treatment for meningioma depends on several factors, including the size and location of the tumor, its growth rate, the patient’s age and overall health, and their goals for treatment [6].

Accurate classification of brain tumors is crucial for determining the appropriate treatment plan. Different imaging techniques can be used to diagnose brain tumors, but MRI is one of the most commonly used non-invasive methods [7]. The popularity of MRI is due to its non-use of ionizing radiation during scanning, its high resolution of soft tissue, and its ability to capture various images using different imaging parameters or enhanced contrast agents [8].

On the other hand, as mentioned earlier; Meningioma can be difficult to diagnose because the tumor is often slow-growing. In addition, the classification step may be confusing and tedious for physicians or radiologists in some complex cases. These require specialists to work on and localize the tumor, compare tumor tissues with adjacent areas, and apply filters to the image if necessary. According to the information above, early diagnosis and accurate classification of meningioma tumor types are vital tasks in evaluation and help choose the most convenient treatment method to save patients’ lives [9].

The process of detecting brain tumors can be quite lengthy, hence the necessity for a Computer-Aided Diagnosis (CAD) system that can detect these tumors at an early stage without any human intervention. Machine Learning (ML) is a specialized field of study that focuses on the development of algorithms and statistical models capable of performing specific tasks by recognizing patterns, without the need for direct instructions [10].

The utilization of conventional machine learning and deep learning techniques in the medical field is a complex task due to the requirement of a large amount of labeled data. Acquiring labeled data is a significant challenge as it involves manual annotation by trained professionals and is often time-consuming and expensive [11].

Also, when the original data representation is high dimensional and the number of examples provided to solve a regression or classification problem is limited, any learning algorithm that does not use prior knowledge will perform poorly due to a lack of data to estimate the model parameters reliably. Due to the increased manual labor required to label data instances, this issue is particularly crucial for applications such as medical image analysis [11]. However, multi-task learning approaches have shown promise in mitigating this issue by leveraging the shared information between several related learning tasks to improve the prediction accuracy of individual tasks [12,13]. By doing so, multi-task learning (MTL) can significantly reduce the dependency on large amounts of labeled data and improve the efficiency of medical data analysis.

Therefore, this research utilizes MRI image data of patients diagnosed with meningioma, based on the aforementioned items. To minimize the requirement for labeled data, multi-task learning models will be employed. These models will incorporate various forms of input data, not just limited to images.

The present study proposes an MTL approach to predict meningioma tumor grade. In this framework, illustrated in Fig 3, the classification of meningioma tumors based on imaging data is considered the primary task. In contrast, the age and gender of patients, gathered in tabular data, are treated as auxiliary tasks. The proposed approach formulates meningioma tumor grade prediction as an MTL problem and leverages the interdependence among multiple tasks to improve the overall prediction accuracy. It offers a promising avenue to advance meningioma tumor classification, a critical step towards personalized medicine, and improved patient outcomes.

## 2. Related work

In recent times, there have been significant improvements in the field of machine learning, which has generated a lot of interest from industry, academia, and popular culture [14]. One of the key drivers of these advancements is the development of artificial neural networks, also known as deep learning. These techniques and algorithms allow computers to identify complex patterns in large sets of data. The progress in machine learning can be attributed to the availability of “big data,” user-friendly software frameworks, and an increase in computing power, enabling deeper neural networks than ever before [15].

Different types of machine learning are classified based on how the models use their input data during training. For example, single-task learning (STL) based on deep learning is the use of deep network models that receive brain images and perform classification as the output goal [16,17].

In recent times, deep learning has demonstrated remarkable performance in the analysis of medical images, particularly in the classification of brain tumors. Compared to classical machine learning techniques, deep learning networks have achieved higher accuracy. The use of CNNs in deep learning has gained significant recognition due to their ability to automatically extract deep features by adapting to even minor changes in images [18–20].

Although deep learning models are capable of accurately classifying data, they rely heavily on a vast amount of labeled data to achieve optimal results. Within the healthcare industry, this poses a challenge for researchers as it requires expert opinions to label each image, resulting in a costly and time-consuming process.

Various solutions have been introduced to address the challenge of limited labeled data. Two popular approaches are multi-task learning and self-supervised learning [11,21]. In this study, the multi-task learning approach was used. This method utilizes additional patient characteristics (age and gender), other than just images, that significantly impact the classification of meningioma tumors [22].

A task is generally defined as learning an output target using a single input source. In this sense, “multiple tasks” can mean learning multiple output goals using a single input source, learning a single output goal using multiple input sources, or a combination of both. Therefore, MTL has three types: a mode with multiple inputs and multiple outputs, a mode with a single input source and several output targets, and finally, a mode with several types of inputs to reach one type of output target [11]. The model used in this research is of this type.

In [23] study, a multi-feature guided convolutional neural network (CNN) architecture is proposed for simultaneous enhancement, segmentation, and classification of bone surfaces from US data.

In [24], a novel deep belief network based multi-task learning algorithm is developed for the classification issue. In particular, the dropout technology and zero-masking strategy are exploited to overcome the overfitting problem and enhance the generalization ability and robustness of the model.

In [25] paper, Li et al. propose a multitask multiscale residual attention network (MMRAN) to simultaneously solve the problem of accurately segmenting and classifying brain tumors. The proposed MMRAN is based on U-Net, and a parallel branch is added at the end of the encoder as the classification network.

Shreyanth et al. (Shreyanth et al. 2023) describe a novel technique for brain tumor segmentation and classification that employs three deep learning architectures: GlobalNet, Multi-task Learning, and FusionNet.

This research project addresses meningioma grade prediction as an MTL problem. This framework considers predicting the type of meningioma tumor from imaging data as the primary task, while demographic data such as age and gender serve as auxiliary tasks. to the best of our knowledge, this type of MTL has not been used for brain tumor classification.

This approach ensures a comprehensive and accurate prediction of meningioma grade, which is crucial for the successful treatment of this disease.

Based on initial evaluations, collaborative learning of patient data leads to significant improvements compared to STL approaches. Among the STL models, the pre-trained DenseNet121 model provides the best result, with a test data accuracy of 76.11%. However, it’s worth noting that the MTL model achieved the best result, with 99.6% accuracy for the test data, as expected.

## 3. Methods

This study was approved by the Research Ethics Committee of Tarbiat Modares University (approval code: IR.MODARES.REC.1402.138). Our study is a retrospective study which analyzes the data collected and archived in the hospital information system previously. Therefore, no patient has participated in this study directly and there is no need for getting informed consent for participation from patients. On the other hand, data records have been anonymized before feeding to us for analysis. Thus, the need for consent to participate was waived by Research Ethics Committee of Tarbiat Modares University according to national regulations.

### 3.1. Meningioma tumor database

The data utilized in this research has been granted approval by the Research Ethics Committee of Tarbiat Modares University through the acceptance code IR.MODARES.REC.1402.138.

This dataset consists of brain MRI images from 34 patients diagnosed with meningioma. The images were obtained from the Mahdia Imaging Center in Hamadan, Iran. The data were accessed on December 2023 for research purposes. The dataset contains a total of 2218 images, each with dimensions of 512x512 and three channels (RGB). Data were accessed for research purposes in 11/01/2024.

The original MRI images are typically acquired as single-channel (grayscale) images. However, the dataset provided by the Mahdia Imaging Center was stored in a 3-channel (RGB) format, with each channel containing identical grayscale information.

**Fig 1:**
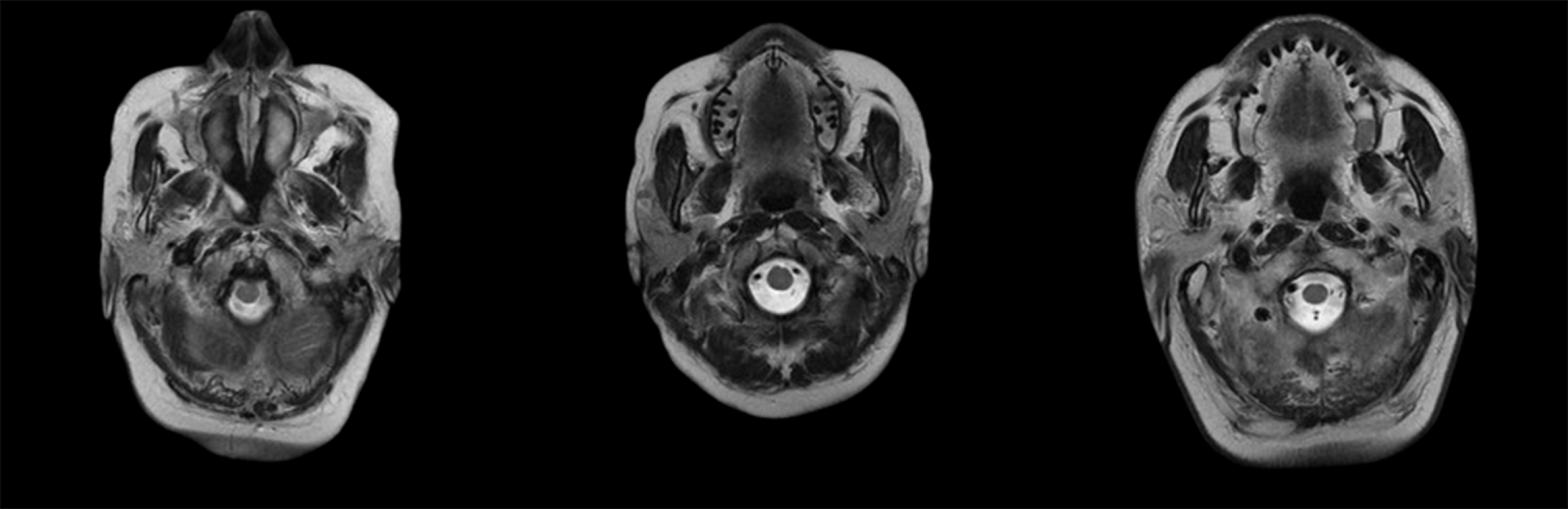
Exemplification of three distinct classes from this study.

The dataset includes 3 class labels representing 3 different degrees of disease. Fig 1 displays 3 sample images from this dataset, each belonging to a different class.

The MRI images presented in Fig 1 incorporate essential information, such as the date of imaging, the patient’s age, and gender. This information is crucial for accurate classification in the MTL approach.

To train both single-task and multi-task supervised models, it is necessary to divide the dataset into 3 subsets for training, validation, and test. The approximate ratio of data in these subsets should be 60%, 12%, and 28% respectively. For this purpose, stratified 6-fold cross-validation is used for splitting original training dataset into training and validation splits.

After splitting the dataset into three subsets – train, test, and validation – data is preprocessed and then, single-task learning models were applied to the data. These models will be discussed in the following section.

Data preprocessing steps are data augmentation with simple operations including shifting, cropping and adding small random noise to images. Moreover, images are normalized with min-max normalization technique. Each image is resized to 224*224 pixels to be compatible with the input size of the pretrained models.

### 3.2. Single task learning with neural networks

In section 2, we discussed single-task learning, a type of machine learning and artificial intelligence. It involves using labeled datasets to train algorithms that classify data or predict outcomes accurately. During the cross-validation process, the model adjusts its weights until it is adequately fitted to the input data.

**Fig 2:**
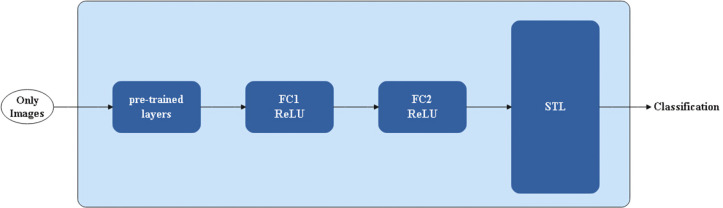
Description of a single-task learning model.

Fig 2 displays an instance of a single-task learning model that takes just an image dataset as input and produces a classification as output, utilizing a pre-trained model.

In the section on transfer learning, we employ three architecture models: MobileNet [26], DenseNet121 [27], and EfficientNetB0 [28].

The approach for feature extraction in both the STL and MTL sections involved training the feature extraction layers with ImageNet weights. After extracting the feature vector, a flattened layer and three fully connected layers with 64, 128, and 3 neurons were used, along with ReLU, ReLU, and Softmax activation functions. The categorical cross-entropy loss function and the Adam optimizer were also utilized.

We utilized MobileNet, DenseNet121, and EfficientNetB0 architectures initialized with ImageNet weights. These models were chosen for their proven efficiency and performance in various image classification tasks.

Initially, the convolutional base of each pre-trained model was frozen, and only the newly added fully connected layers (with 64, 128, and 3 neurons) were trained on our MRI dataset.

After initial convergence, we unfroze the last few layers of the convolutional base and continued training with a reduced learning rate (1e-5) to allow the model to adapt more specifically to MRI features, as recommended in medical image transfer learning literature [Tajbakhsh et al., 2016].

To further facilitate domain adaptation and reduce overfitting, we applied augmentation techniques such as random rotations, flips, and adding small random noise during training. However, we acknowledge that natural images (ImageNet) differ significantly from medical MRI images, and a domain shift issue occurs.

Section 4 will mention the results of all these models and compare the performance of these architectures with the multi-task learning approach in detail.

### 3.3. Multi task learning with neural networks

The focus of this research is to develop a multitask learning model that will aid in the prediction of meningioma tumor type through imaging data. The model aims to simultaneously learn the primary task of predicting the tumor type, while also considering the auxiliary tasks of age and gender. The inclusion of these auxiliary tasks is expected to improve the accuracy of the model’s predictions. Our model does not currently include a segmentation branch, and the tasks are handled in parallel after shared feature extraction from the imaging data.

To achieve this objective, the model uses a dataset that comprises both imaging data and epidemiological data. The imaging data includes MRI of patients with meningioma tumors, while the epidemiological data includes the patient’s age and gender information.

**Fig 3:**
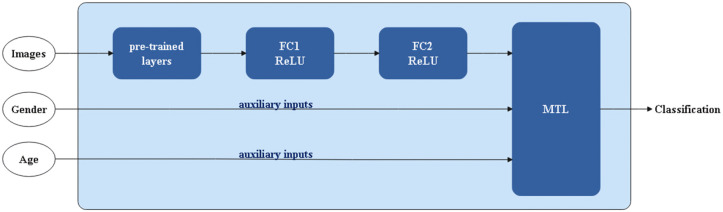
Multi-task learning for predicting the grade of meningiomas.

Fig 3 shows the general trend of the multitask learning model used in this research. The model first processes the imaging data to extract relevant features that are then used to predict the tumor type. Simultaneously, the model processes the age and gender data to learn additional features that aid in the prediction of the tumor type.

Overall, this research aims to develop a multitask learning model that can accurately predict the type of meningioma tumor using both imaging and epidemiological data.

The MTL architecture includes:

- Feature Extraction: MRI images are processed through a convolutional neural network (CNN) backbone (e.g., EfficientNetB0) to extract deep imaging features.
- Demographic Data Processing: Age (numerical, normalized) and gender (binary, one-hot encoded) are input as auxiliary features. These are passed through a small fully connected (dense) layer to create a demographic feature embedding.
- Feature Fusion: The image feature vector and the demographic embedding are concatenated to form a joint representation. This joint vector is then used as input to three parallel output heads:
- Primary head: Classifies tumor grade (3-class softmax).
- Auxiliary head 1: Predicts patient age (regression).
- Auxiliary head 2: Predicts patient gender (binary classification).
- Loss Function: The total loss is a weighted sum of the categorical cross-entropy (tumor grade), mean squared error (age), and binary cross-entropy (gender).

We acknowledge that, to date, age and gender are not established as independent predictors for meningioma grade classification in clinical guidelines. However, several studies suggest that according to epidemiological trends, meningiomas are more common in females, and certain subtypes or grades may have age-related prevalence (Wiemels et al., 2010; Claus et al., 2012).

Moreover, as prognostic factors, age and gender are often included as covariates in outcome studies, though their direct role in grade classification is limited.

Therefore, in our study, age and gender are used as auxiliary tasks in the MTL framework, not as direct predictors of tumor grade. The purpose is to encourage the model to learn more generalizable and robust imaging features by leveraging demographic context, which can act as a regularizer and improve overall learning efficiency-particularly with small datasets (Ruder, 2017).

However, the inclusion of age and gender is primarily a methodological strategy to enhance model generalization and is not meant to suggest clinical causality or replace established diagnostic criteria. Therefore, we explicitly acknowledge this limitation and call for caution in interpreting the clinical significance of these auxiliary tasks.

## 4. Results and discussion

### 4.1. Results of STL methods

In the previous section, specifically section 3.2, we employed three pre-trained models, MobileNet, DenseNet121, and EfficientNetB0, to conduct a comparative analysis of the performance of two distinct approaches: STL and MTL. In this section, we will focus on evaluating the effectiveness of the single-task learning approach.

The hyperparameters of each model are chosen with Grid search method and the best hyperparameters are learning rate of 0.001, optimizer of ‘Adam’, drop-out rate of 0.5 and the models are trained for 100 epochs with batch size of 64.

**Fig 4:**
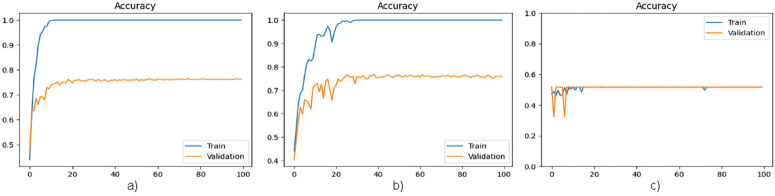
a) Graph of accuracy per epoch of MobileNet architecture. **b)** Graph of accuracy per epoch of DenseNet121 architecture. **c)** Graph of accuracy per epoch of EfficientNetB0 architecture.

**Fig 5:**
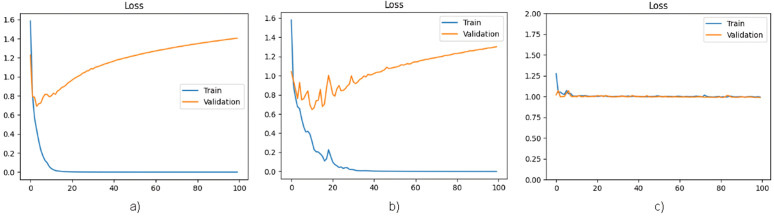
a) Graph of loss function per epoch of MobileNet architecture. **b)** Graph of loss function per epoch of DenseNet121 architecture. **c)** Graph of loss function per epoch of EfficientNetB0 architecture.

Figs 4 and 5 present a graphical representation of the accuracy and loss function per epoch, respectively, for all three architectures.

The results presented in Figs 4 and 5 indicate that the MobileNet and DenseNet121 architectures exhibit overfitting in the STL approach, where the models receive only images as input. This observation highlights the requirement for additional data samples to ensure reliable learning. Furthermore, the analysis reveals that the architecture of EfficientNetB0 is underfitted, necessitating a resolution to enhance its performance.

In order to conduct a comprehensive performance analysis of the three aforementioned architectures, additional evaluation criteria based on macro average have been presented in Table 1.

**Table 1:** Comparison of evaluation criteria of three architectures used in the STL approach.

	Accuracy	Precision	Recall	f1-score
MobileNet	0.74	0.75	0.72	0.73
DenseNet121	0.76	0.77	0.76	0.77
EfficientNetB0	0.51	0.17	0.33	0.23

### 4.2. Results of MTL methods

The findings of the single-task learning method have highlighted the need to improve the accuracy of models when classifying brain MRI images for meningioma tumor diagnosis and classification. The solution to this would be to acquire more images, but as previously noted in Section 1, the health industry faces the challenge of a shortage of labeled images.

As a result, we have endeavored to boost the performance of the three architectures employed in the single-task learning method in this section. We achieved this by incorporating other patient characteristics that are directly linked to meningioma tumor classification, such as age and gender. We accomplished this without the use of data augmentation or the need for additional image data.

**Fig 6:**
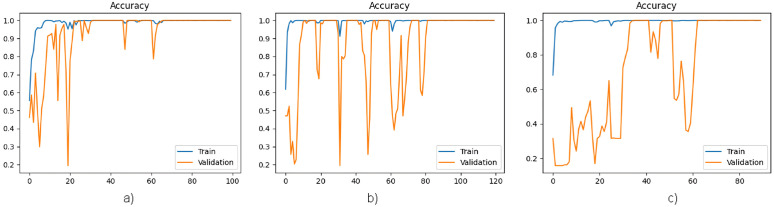
a) Graph of accuracy per epoch of MobileNet architecture. **b)** Graph of accuracy per epoch of DenseNet121 architecture. **c)** Graph of accuracy per epoch of EfficientNetB0 architecture.

**Fig 7:**
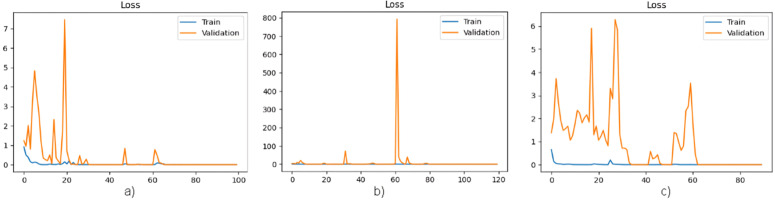
a) Graph of loss function per epoch of MobileNet architecture. **b)** Graph of loss function per epoch of DenseNet121 architecture. **c)** Graph of loss function per epoch of EfficientNetB0 architecture.

In the following, Figs 6 and 7, respectively, depict the accuracy and loss function per epoch graphs for three distinct architectures employed in conjunction with an MTL approach.

The analysis of Figs 4–7 reveals that the inclusion of patient characteristics in each image has effectively addressed the overfitting challenge that was previously encountered by the MobieNet and DenseNet121 architectures. Furthermore, this approach has also resolved the underfitting issue that was observed in the EfficientNetB0 architecture. These findings suggest that incorporating patient-level features has a significant impact on the performance of image classification models, thereby enhancing their predictive capabilities.

**Fig 8:**
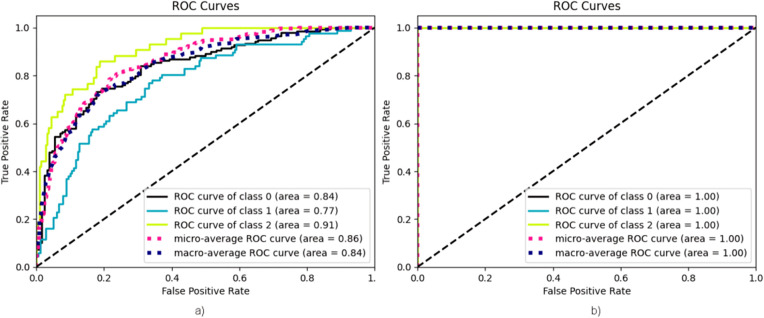
a) ROC diagram of MobileNet architecture in the single-task learning approach. **b)** ROC diagram of MobileNet architecture in the multi-task learning approach.

It is widely recognized that in the multitasking learning approach, all architectures achieved the utmost accuracy, with evaluation criteria for each of the three architectures reaching 99.6% ± 0.2 in 95% confidence interval. Fig 8 displays an ROC diagram that compares the performance of the MobileNet architecture in both single-task and multi-task learning approaches against one another.

MTL reduces overfitting by introducing auxiliary tasks (age and gender prediction) that act as additional regularizers. This forces the shared representation layers to learn more generalizable features rather than over-specializing to the primary classification task. As a result, the model is less likely to memorize noise or patient-specific artifacts present in the limited dataset.

However, we acknowledge that the dataset size (34 patients) is a significant limitation. Deep learning models typically require much larger and more diverse datasets to generalize well. The main reason for using pre-trained CNNs is the small number of images. Therefore, further validation on larger, multi-center datasets is necessary in future studies. We now emphasize the need for future studies with larger, more diverse, and multi-institutional datasets to validate the proposed approach and ensure clinical applicability.

Moreover, we have implemented stratified 6-fold cross-validation, ensuring that each fold contains unique patients. This provides a more robust estimate of model performance and helps mitigate the risk of overfitting to intra-patient variations.

On the other hand, to avoid overfitting, regularization techniques, including dropout and early stopping, are used.

Also, while our multi-task learning approach demonstrated promising technical performance in retrospective analysis, it is important to note that our study did not include clinical trials, prospective validation, or integration into real-world clinical workflows. As such, any claims regarding the use of this model in computer-aided diagnostic systems remain speculative. Further validation using larger, multi-center datasets and evaluation within clinical settings are necessary to establish the model’s true clinical utility and generalizability.

## 5. Conclusion and future works

The aim of this study was to develop a multi-task learning model that enhances the precision of prior and single-task approaches in brain image classification, specifically in meningioma tumor classification. This was done by utilizing MRI images in conjunction with patient gender and age characteristics as inputs for deep learning models.

While using ImageNet pre-trained models like MobileNet and DenseNet121 can accelerate training and improve initial convergence for medical imaging tasks, this approach has important limitations due to the significant domain gap between natural images and medical MRI scans. Features learned from natural images may not optimally capture the subtle patterns relevant to medical diagnosis, and the replication of grayscale MRI images into three RGB channels does not add meaningful information. As a result, model generalization and robustness may be suboptimal, especially with small, specialized datasets. Recent research suggests that self-supervised learning (SSL) or domain-specific pre-training on large collections of unlabeled or medical images can yield more relevant feature representations, enhance model performance, and better address the unique challenges of medical image analysis. Therefore, as future work, we propose to explore SSL and domain-specific pre-training strategies to further improve the accuracy and clinical utility of meningioma tumor classification models.

This research aims to facilitate medical practitioners’ accurately and expeditiously forecasting the classification of meningioma tumors. The proposed methodology serves as an effective preliminary assessment tool for healthcare professionals, offering insightful information for clinical decision-making in pre-treatment and surgical evaluation.

Although our proposed method shows potential for supporting computer-aided diagnosis, additional clinical studies and real-world validation are required before it can be recommended for actual use in diagnostic systems.

Acquiring large amounts of labeled data is a major challenge in medical data analysis and modeling, but it’s necessary for developing accurate models. This research has shown outstanding performance in this area by using age and gender features that are directly linked to tumor grade, as well as combining images and tabular features. This research has opened up new possibilities for future studies to reduce the need for large amounts of labeled data, making a significant contribution to the field of medical data analysis and modeling.

The main task of the multi-task learning model used in this research is to predict the tumor grade, while the patients’ age and gender act as auxiliary tasks. Notably, all three architectures pre-trained through the proposed MTL approach demonstrate superior performance compared to their counterparts in single-task learning for meningioma tumor classification.

Thanks to advancements in computing power, there are now alternative imaging techniques available for future MRI studies. Furthermore, multi-task learning can be applied to broaden the range of inputs and outputs in the model – such as segmentation and classification goals – resulting in improved accuracy in brain tumor segmentation. This, in turn, aids in precise diagnosis and treatment planning by providing a comprehensive and accurate tumor image. By leveraging these methods, specialists can make informed decisions on the most effective treatment approach.

Finally, future works could extend the architecture to incorporate a dedicated segmentation branch, leveraging the synergy between segmentation and classification for improved performance, as demonstrated in recent literature.

## References

[1] DeAngelis LM. Brain tumors. N Engl J Med. 2001;344(2):114–23. doi: 10.1056/NEJM200101113440207 11150363

[2] Behin A, Hoang-Xuan K, Carpentier AF, Delattre J-Y. Primary brain tumours in adults. Lancet. 2003;361(9354):323–31. doi: 10.1016/S0140-6736(03)12328-8 12559880

[3] Gittleman HR, Ostrom QT, Rouse CD, Dowling JA, de Blank PM, Kruchko CA, et al. Trends in central nervous system tumor incidence relative to other common cancers in adults, adolescents, and children in the United States, 2000 to 2010. Cancer. 2015;121(1):102–12. doi: 10.1002/cncr.29015 25155924 PMC4298242

[4] Walker EV, Davis FG, CBTR founding affiliates. Malignant primary brain and other central nervous system tumors diagnosed in Canada from 2009 to 2013. Neuro Oncol. 2019;21(3):360–9. doi: 10.1093/neuonc/noy195 30649461 PMC6380406

[5] Gerstner ER, Batchelor TT. Primary central nervous system lymphoma. Primary Central Nervous System Tumors: Pathogenesis and Therapy. 2011. 333–53.

[6] Huang RY, Bi WL, Griffith B, Kaufmann TJ, la Fougère C, Schmidt NO, et al. Imaging and diagnostic advances for intracranial meningiomas. Neuro Oncol. 2019;21(Suppl 1):i44–61. doi: 10.1093/neuonc/noy143 30649491 PMC6347083

[7] Sultan HH, Salem NM, Al-Atabany W. Multi-Classification of Brain Tumor Images Using Deep Neural Network. IEEE Access. 2019;7:69215–25. doi: 10.1109/access.2019.2919122

[8] Litjens G, Kooi T, Bejnordi BE, Setio AAA, Ciompi F, Ghafoorian M, et al. A survey on deep learning in medical image analysis. Med Image Anal. 2017;42:60–88. doi: 10.1016/j.media.2017.07.005 28778026

[9] Louis DN, Perry A, Reifenberger G, von Deimling A, Figarella-Branger D, Cavenee WK, et al. The 2016 World Health Organization Classification of Tumors of the Central Nervous System: a summary. Acta Neuropathol. 2016;131(6):803–20. doi: 10.1007/s00401-016-1545-1 27157931

[10] Bishop CM, Nasrabadi NM. Pattern recognition and machine learning. Springer. 2006.

[11] Thung K-H, Wee C-Y. A brief review on multi-task learning. Multimed Tools Appl. 2018;77(22):29705–25. doi: 10.1007/s11042-018-6463-x

[12] Vlachostergiou A, Tagaris A, Stafylopatis A, Kollias S. Multi-task learning for predicting Parkinson’s disease based on medical imaging information. In: 2018 25th IEEE International Conference on Image Processing (ICIP), 2018.

[13] Huang H, Yang G, Zhang W, Xu X, Yang W, Jiang W, et al. A Deep Multi-Task Learning Framework for Brain Tumor Segmentation. Front Oncol. 2021;11:690244. doi: 10.3389/fonc.2021.690244 34150660 PMC8212784

[14] Rana M, Bhushan M. Machine learning and deep learning approach for medical image analysis: diagnosis to detection. Multimed Tools Appl. 2022;:1–39. doi: 10.1007/s11042-022-14305-w 36588765 PMC9788870

[15] Lundervold AS, Lundervold A. An overview of deep learning in medical imaging focusing on MRI. Z Med Phys. 2019;29(2):102–27. doi: 10.1016/j.zemedi.2018.11.002 30553609

[16] LeCun Y, Bengio Y, Hinton G. Deep learning. Nature. 2015;521(7553):436–44. doi: 10.1038/nature14539 26017442

[17] Kim HE, Cosa-Linan A, Santhanam N, Jannesari M, Maros ME, Ganslandt T. Transfer learning for medical image classification: a literature review. BMC Med Imaging. 2022;22(1):69. doi: 10.1186/s12880-022-00793-7 35418051 PMC9007400

[18] Özcan H, Emiroğlu BG, Sabuncuoğlu H, Özdoğan S, Soyer A, Saygı T. A comparative study for glioma classification using deep convolutional neural networks. Molecular Biology and Evolution. 2021. 10.3934/mbe.2021080 33757198

[19] Tripathi PC, Bag S. A computer-aided grading of glioma tumor using deep residual networks fusion. Comput Methods Programs Biomed. 2022;215:106597. doi: 10.1016/j.cmpb.2021.106597 34974232

[20] Nayak DR, Padhy N, Mallick PK, Zymbler M, Kumar S. Brain Tumor Classification Using Dense Efficient-Net. Axioms. 2022;11(1):34. doi: 10.3390/axioms11010034

[21] Huang S-C, Pareek A, Jensen M, Lungren MP, Yeung S, Chaudhari AS. Self-supervised learning for medical image classification: a systematic review and implementation guidelines. NPJ Digit Med. 2023;6(1):74. doi: 10.1038/s41746-023-00811-0 37100953 PMC10131505

[22] Alruwaili AA, De Jesus O. Meningioma. StatPearls [Internet]. StatPearls Publishing. 2022. 32809373

[23] Wang P, Patel VM, Hacihaliloglu I. Simultaneous segmentation and classification of bone surfaces from ultrasound using a multi-feature guided CNN. In: Medical Image Computing and Computer Assisted Intervention–MICCAI 2018: 21st International Conference, Granada, Spain, September 16-20, 2018, Proceedings, Part IV, 2018.

[24] Zeng N, Li H, Peng Y. A new deep belief network-based multi-task learning for diagnosis of Alzheimer’s disease. Neural Comput & Applic. 2021;35(16):11599–610. doi: 10.1007/s00521-021-06149-6

[25] Li G, Hui X, Li W, Luo Y. Multitask Learning with Multiscale Residual Attention for Brain Tumor Segmentation and Classification. Mach Intell Res. 2023;20(6):897–908. doi: 10.1007/s11633-022-1392-6

[26] Howard AG, Zhu M, Chen B, Kalenichenko D, Wang W, Weyand T, et al. Mobilenets: Efficient convolutional neural networks for mobile vision applications. arXiv preprint. 2017. doi: 10.48550/arXiv.1704.04861

[27] Huang G, Liu Z, Van Der Maaten L, Weinberger KQ. Densely connected convolutional networks. In: Proceedings of the IEEE Conference on Computer Vision and Pattern Recognition, 2017.

[28] Tan M, Le Q. Efficientnet: Rethinking model scaling for convolutional neural networks. In: International conference on machine learning, 2019.

